# The synergistic effect of homocysteine and lipopolysaccharide on the differentiation and conversion of raw264.7 macrophages

**DOI:** 10.1186/1476-9255-11-13

**Published:** 2014-05-13

**Authors:** Shanshan Gao, Lijun Wang, Weimin Liu, Yue Wu, Zuyi Yuan

**Affiliations:** 1 Department of Cardiology, the First Affiliated Hospital of Medical College, Xi’an Jiaotong University, Xi’an 710061, China

**Keywords:** Homocysteine, Lipopolysaccharide, Macrophages, M1/M2 subtype, Polarization, Subtype conversion

## Abstract

**Background:**

Macrophages play pivotal roles in the progression of atherosclerosis (AS) and their heterogeneous differentiation patterns have been studied extensively. The classical subtype of activated macrophage, M1, promotes the progression of AS. Conversely, the alternative subtype of activated macrophage, M2, is regarded as a repressor of AS. Homocysteine (Hcy) may influence macrophage subtype polarization both in vivo and in vitro. Homocysteinemia (HHcy) is an independent risk factor in coronary heart disease and the effect of Hcy on macrophage differentiation has not been studied until now.

**Methods:**

Different concentrations of Hcy in combination with a fixed concentration of lipopolysaccharide (LPS, 200 ng/mL) were used to treat RAW264.7 macrophages. Real-time PCR was used to detect and quantify RNA transcripts indicative of M1 and M2 differentiation. The efficacy and specificity for each chemical stimulant in inducing macrophage differentiation were also investigated. The M2 macrophages (anti-inflammatory subtype) induced using classical methods (IL-4, 10 ng/mL) were also treated with different concentrations of Hcy complemented with LPS. The synergistic effect of Hcy and LPS in the converting the M2 subtype to M1 was also studied.

**Results:**

Macrophages can be induced to differentiate towards M1 by a combination of Hcy with LPS, with the strongest effect observed at an Hcy concentration of 50 μmol/L. After inducing macrophages to the M2 subtype using IL-4, treatment with both Hcy and LPS could elicit conversion from the M2 to M1 subtype.

**Conclusion:**

Combined treatment with Hcy and LPS can induce the polarization of cultured RAW264.7 macrophages into the pro-inflammatory subtype, as well as promote subtype conversion from anti-inflammatory to pro-inflammatory.

## Background

Atherosclerosis (AS) is a chronic and progressive inflammatory disease characterized by the accumulation of lipids and fibrous lesions in the large arterial intima. Mechanisms governing AS continue to draw extensive research interest. Among proposed mechanisms, the immuno-inflammation hypothesis has become accepted by most researchers [1-3]. Macrophages are important in both the initiation and progression of AS [4,5]. Macrophages can differentiate into two subtypes (M1 and M2) in response to micro-environmental changes. Considered the classical activated subtype, M1 exerts pro-inflammatory effects that promote the progression of AS. In contrast, the M2 activated subtype exerts anti-inflammatory effects and is regarded as a repressor of AS [6,7].

Hyperhomocysteinemia (HHcy, abnormally high physiological levels of homocysteine, Hcy) is regarded as an independent risk factor in coronary heart disease, although the exact mechanism of how Hcy induces AS is unclear. Studies have shown that Hcy can promote AS occurrence and progression through oxidative stress damage, endothelial damage, and activation of inflammatory and immune responses [8,9]. A positive correlation between HHcy, endothelial dysfunction, and accelerated AS has been demonstrated by using gene-induced and diet-induced HHcy animal models [10]. The results of some studies also reveal that Hcy could induce monocyte/macrophage localization in inflammatory lesions and exert pro-AS effects. Additionally, researchers have found that Hcy can influence the differentiation of macrophages both in vivo and in vitro [11]. However, it is unclear if the effect of Hcy on macrophage polarization and subtype conversion correlates to its pathogenic role in AS.

## Materials and methods

### Cell culture

The RAW264.7 macrophage cell line was provided by the Cardiology Institute of the First Affiliated Hospital of Medical College, Xi’an Jiaotong University [12]. RAW264.7 cells were cultured in DMEM supplemented with 10% (vol/vol) FBS, penicillin (100 U/mL) and streptomycin (100 μg/mL). In a 6-well round bottom plate, 1 × 10^6^ cells per well were seeded and incubated at 37°C for 12 hours. Before stimulation by Hcy and LPS, cells were serum-starved by culturing in DMEM without 10% FBS for 12 hours.

### Reagents

Hcy and LPS were from Sigma (Milwaukee, USA). IL-4 was from Peprotech (Rocky Hill, USA). The upstream and downstream primers of GAPDH, IL-6, IL-10, TNF-α, iNOS, Arg-1, and MMR (shown in Table 1) were synthesized by Beijing Augct Biotechnology Co., Ltd. (Beijing, China). The RNA Fast 200 kit was from Shanghai Flytech Biotechnology Co., Ltd (Shanghai, China). The RevertAid™ First Strand cDNA Synthesis Kit was from Fermentas (Vilnius, Lithuania). SYBR *Premix Ex Taq*™ II was from TaKaRa (Dalian, China).

**Table 1:** Sequences of real-time PCR primers

**Genes**	**Primers**	
GAPDH	F (5′-3′)	TCA ACG GCA CAG TCA AGG
	R (5′-3′)	ACT CCA CGA CAT ACT CAG C
IL-6	F (5′-3′)	AGC CAG AGT CCT TCA GAG AGA TAC
	R (5′-3′)	AAT TGG ATG GTC TTG GTC CTT AGC
TNF-α	F (5′-3′)	GCT CTT CTG TCT ACT GAA CTT CGG
	R (5′-3′)	ATG ATC TGA GTG TGA GGG TCT GG
iNOS	F (5′-3′)	CCC TTC CGA AGT TTC TGG CAG CAG C
	R (5′-3′)	CCA AAG CCA CGA GGC TCT GAC AGC C
IL-10	F (5′-3′)	GGT TGC CAA GCC TTA TCG TA
	R (5′-3′)	ACC TGC TCC ACT GCC TTG CT
Arg-1	F (5′-3′)	ATG CTC ACA CTG ACA TCA ACA CTC
	R (5′-3′)	CTC TTC CAT CAC CTT GCC AAT CC
MMR	F (5′-3′)	CAT GAG GCT TCT CCT GCT TCT G
	R (5′-3′)	TTG CCG TCT GAA CTG AGA TGG

F: forward primer; R: reverse primer.

### RNA isolation and real-time PCR

Total RNA was isolated with the RNA Fast 200 kit according to the manufacturer’s instructions. First-strand cDNA was synthesized with the RevertAid™ First Strand cDNA Synthesis Kit and analyzed by real-time quantitative PCR with SYBR *Premix Ex Taq*™ II. PCR was performed on the IQ5™ Multicolor real-time PCR and detection system (Bio-Rad, Hercules, CA, USA). Data were analyzed on the basis of relative expression method using the formula ΔΔC expression = 2^–ΔΔCt^, where ΔΔCt = ΔCt (treated group) – ΔCt (control group), ΔCt = Ct (target gene) – Ct (GAPDH), and Ct = cycle at which the threshold is reached.

### Statistical analysis

Data were analyzed with SPSS for Windows (version 18.0) using one-way ANOVA. Unless otherwise stated, graphs show mean ± SD. For each dataset, *p* < 0.05 was considered statistically significant.

## Results

### Effects of Hcy and LPS on mRNA expression in macrophages

**Figure 1:**
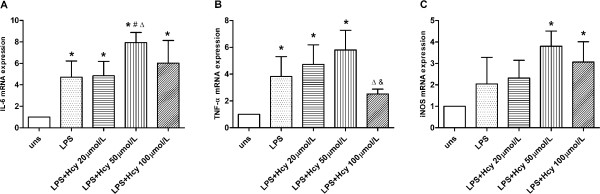
**Effects of Hcy and LPS on mRNA expression of M1 marker genes. (A)** IL-6, **(B)** TNF-α and **(C)** iNOS mRNA expression at 12 h were assessed by real-time PCR. Gene expression is represented as fold-change compared to unstimulated macrophage. Data are representative of three independent experiments. (uns: unstimulated macrophages; *p < 0.05 *vs* uns, #*p* < 0.05 *vs* LPS, Δ*p* < 0.05 *vs* LPS + Hcy 20 μmol/L, &*p* < 0.05 *vs* LPS + Hcy 50 μmol/L).

Different concentrations of Hcy (0, 20, 50 and 100 μmol/L) combined with a fixed concentration of LPS (200 ng/mL) were tested. The mRNA levels of M1 markers (IL-6, TNF-α, and iNOS) were up-regulated by the 12-hour treatment with different concentrations of Hcy combined with LPS. mRNA expression levels of these genes reached their maxima at an Hcy concentration of 50 μmol/L (Figure 1). Expression of M2 marker genes did not change significantly in response to the combined Hcy/LPS treatment (data not shown).

### Time effect of Hcy and LPS treatment on mRNA expression in macrophages

**Figure 2:**
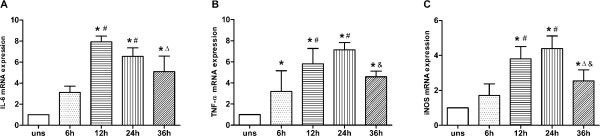
**Time effect of Hcy and LPS on mRNA expression of M1 markers genes. (A)** IL-6, **(B)** TNF-α and **(C)** iNOS mRNA expression were assessed by real-time PCR. Gene expression is represented as fold-change compared to unstimulated macrophage. Data are representative of three independent experiments. (uns: unstimulated macrophages; *p < 0.05 *vs* uns, #*p* < 0.05 *vs* 6 h, Δ*p* < 0.05 *vs* 12 h, &*p* < 0.05 *vs* 24 h).

M1-associated genes reached their highest mRNA expression levels at an Hcy concentration of 50 μmol/L. To determine if Hcy affects expression of M1-associated genes in a time-dependent manner, we measured the corresponding mRNA transcript levels at different time points. When cells were treated with 50 μmol/L Hcy and 200 ng/mL LPS, mRNA expression of IL-6, TNF-α, and iNOS peaked after 12, 24, and 24 hours, respectively (Figure 2).

### Effects of Hcy and LPS on mRNA expression in M2 macrophages

**Figure 3:**
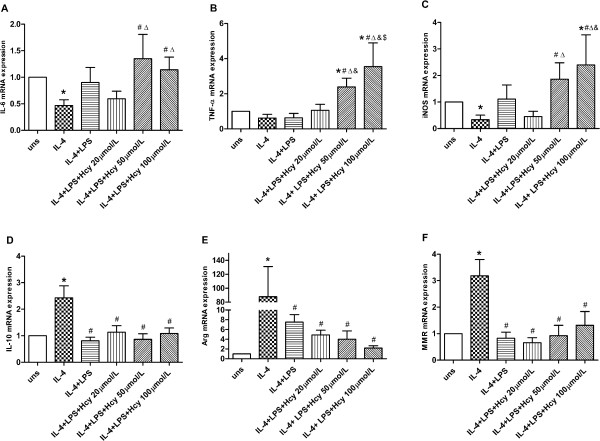
**Effects of Hcy and LPS on mRNA expression in M2 macrophage. (A)** IL-6, **(B)** TNF-α, **(C)** iNOS, **(D)** IL-10, **(E)** Arg-1 and **(F)** MMR mRNA expression were assessed by real-time PCR. Gene expression is represented as fold-change compared to unstimulated macrophages. Data are representative of three independent experiments. (uns: unstimulated macrophages; Arg stands for Arg-1; *p < 0.05 *vs* uns; #*p* < 0.05 *vs* IL-4; Δ*p* < 0.05 *vs* LPS; &*p* < 0.05 *vs* LPS + Hcy 20 μmol/L; $*p* < 0.05 *vs* LPS + Hcy 50 μmol/L).

Because the combination of Hcy and LPS could induce macrophage differentiation towards the M1 subtype, we were compelled to investigate if similar treatment could convert the M2 subtype into M1. RAW264.7 macrophages were first induced into the M2 subtype using IL-4 (10 ng/mL) for 12 hours. The three M2 markers (IL-10, Arg-1, and MMR) were up-regulated, whereas the M1 markers (IL-6, TNF-α, and iNOS) were down-regulated, indicating the successful polarization to M2. Subsequently, these M2 cell were co-cultured with different concentrations of Hcy and a fixed concentration for 12 hours. After treatment, the gene expression of M2 markers decreased, whereas the gene expression of M1 markers increased (Figure 3).

### Time effect of Hcy and LPS on mRNA expression in M2 macrophages

**Figure 4:**
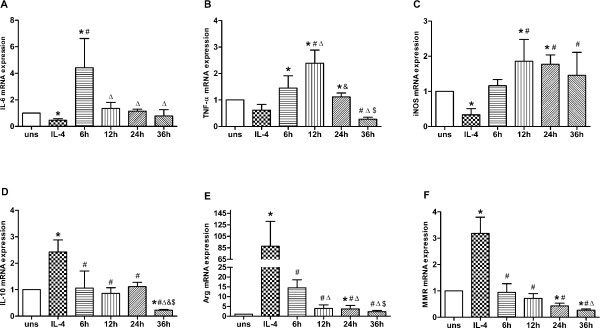
**Time effect of Hcy and LPS on mRNA expression in M2 macrophage. (A)** IL-6, **(B)** TNF-α, **(C)** iNOS, **(D)** IL-10, **(E)** Arg-1 and **(F)** MMR mRNA expression were assessed by real-time PCR. Gene expression is represented as fold-change compared to unstimulated macrophages. Data are representative of three independent experiments. (uns: unstimulated macrophages; Arg stands for Arg-1; *p < 0.05 *vs* uns; #*p* < 0.05 *vs* 6 h; Δ*p* < 0.05 *vs* 12 h; &*p* < 0.05 *vs* 24 h).

To find out whether the effect of combined Hcy and LPS treatment on mRNA expression in M2 macrophages is time-dependent, a combination of 50 μmol/L Hcy and LPS (200 ng/mL) was used to treat M2 macrophages arising from IL-4 induction. mRNA expression levels of IL-6, TNF-α, and iNOS reached their maxima after 6, 12, and 12 hours, respectively. In contrast, the expression of IL-10, Arg-1, and MMR declined after prolonged incubation with the combined treatment (Figure 4).

## Discussion

Macrophages are considered to be important immune effector cells. Recent studies have uncovered plasticity and heterogeneity in their differentiation pathways, as demonstrated by changes in cellular phenotype and physiology in response to micro-environmental stimuli. Mirroring the Th1/Th2 nomenclature, polarized macrophages are categorized into M1 and M2 subtypes. The classical subtype of activated macrophages, M1, can initiate adaptive immune responses and produce various pro-inflammatory cytokines. These biochemical responses are often involved in killing microorganisms and tumor cells. Moreover, M1 macrophages can promote atherosclerosis (AS). The alternative activated subtype, M2, also plays various roles, such as directing Th2 humoral response, killing parasites, repairing tissues, and producing anti-inflammatory cytokines. M2 macrophages are regarded as inhibitors of AS. However, it is noteworthy that the distinction between M1 and M2 macrophages may not be absolutely definitive; many researchers consider the M1 and M2 subtypes as opposing extremes of the macrophage spectrum [13].

The classical activation of M1 macrophages by interferon-γ (IFN-γ) and LPS creates cells that can produce various pro-inflammatory chemokines and cytokines such as IL-6 and TNF-α. M2 macrophages, usually activated by IL-4, produce anti-inflammatory cytokines such as IL-10. Aside from their differential expression of opposing effector molecules, inducible nitric oxide synthase (iNOS) is expressed more in M1 macrophages, whereas arginase-1 (Arg-1) and mannose membrane receptor (MMR) are expressed more in M2 macrophages, reflecting the different immunological roles of these two populations [7]. In this study, we chose IL-6, TNF-α, and iNOS as M1 macrophage markers, whose expression was induced by IFN-γ and LPS. We chose IL-10, Arg-1, and MMR as M2 macrophage markers, whose expression was induced by IL-4. These results are consistent with our previous work [12].

LPS, the primary glycolipid component found in the outer membranes of Gram-negative bacteria, can induce inflammatory responses, activate the complement system, and initiate the coagulation pathway [14]. Many studies have confirmed that in the presence of IL-1 or IFN-γ, the LPS-induced defense reaction and killing ability could be augmented. Conversely, the pro-inflammatory effect of IFN-γ can be enhanced by the presence of LPS [15,16]. Because we had been unable to stimulate macrophage subtype polarization using Hcy alone (data not shown), we decided to use a combination of Hcy and LPS.

In the presence of LPS, Hcy led to up-regulated expression of M1 macrophage markers (IL-6, TNF-α, and iNOS); the optimal Hcy concentration was 50 μmol/L. However, Hcy and LPS elicited no significant effects on the expression of M2 macrophage markers (IL-10, Arg-1, and MMR). No correlation between the concentration of Hcy and the expression of marker genes was observed. This was possibly due to higher Hcy concentrations leading to more cell death. We also found that a combination of Hcy and LPS could transform M2 subtype into M1. This phenomenon may help to explain the pro-atherosclerotic effects of Hcy.

Many studies have shown that Hcy can act as a promoter of AS by activating inflammatory immune reactions. The immuno-inflammation hypothesis is a generally accepted explanation for the mechanism of AS, and macrophages are believed to be important in this process. The differentiation of macrophages into different sub-populations has become a new exploratory area in the initiation and progression of AS. Zhang *et al.* have demonstrated that Hyperhomocysteinemia (HHcy) could accelerate AS and enhance Ly-6C Monocyte/Macrophage accumulation in the lesions of Tg-hCBS apoE^−/−^Cbs^−/−^ mice. They also found that monocyte/macrophage accumulation in the lesions of Ly-6C-positive mice correlated positively with Hcy levels. However, little is known about the influence of Hcy on the polarization of cultured macrophages [17]. In this study, we used a cell-based assay to demonstrate that Hcy could induce macrophage polarization into the pro-inflammatory phenotype, thereby providing further evidence in support of the model for Hcy-induced AS.

## Conclusions

We investigated the synergistic effects of Hcy and LPS on the differentiation and conversion of RAW264.7 macrophages. The results showed that a combination of Hcy and LPS could induce macrophages to differentiate into the pro-inflammatory subtype. The differentiated anti-inflammatory subtype could also be converted to the pro-inflammatory subtype by combined treatment with Hcy and LPS. These findings provide new evidence for the role of Hcy in facilitating the progression of AS.

## Abbreviations

AS: Atherosclerosis; Hcy: Homocysteine; HHcy: Hyperhomocysteinemia; IL-6: Interleukin-6; IL-10: Interleukin-10; LPS: Lipopolysaccharide; iNOS: Inducible nitric oxide synthase; IFN-γ: Interferon-γ; Arg-1: Arginase-1; MMR: Mannose membrane receptor.

## Competing interests

The authors declare that they have no competing interests.

## Authors’ contributions

SG carried out the main body of this work and drafted the manuscript. WL, YW and LW participated in designing the studies. ZY participated in the design and coordination and helped to draft the manuscript. All authors have read and approved the final manuscript.
